# The effects of bioactive components from the rhizome of *Salvia miltiorrhiza* (Danshen) on the characteristics of Alzheimer’s disease

**DOI:** 10.1186/s13020-019-0242-0

**Published:** 2019-05-21

**Authors:** Cheong-Meng Chong, Huanxing Su, Jin-Jian Lu, Yitao Wang

**Affiliations:** State Key Laboratory of Quality Research in Chinese Medicine, Institute of Chinese Medical Sciences, University of Macau, Macao, China

**Keywords:** Alzheimer’s disease, Danshen, Amyloid β plaques, Neurofibrillary tangles, Mitochondrial dysfunction, Autophagy

## Abstract

Alzheimer’s disease (AD) is a common human neurodegenerative disease, which is characterized by the progressive loss of memory and the cognitive impairment. Since the etiology of AD is still unknown, it is extremely difficult to develop the effective drugs for preventing or slowing the AD process. The major characteristics of AD such as amyloid β plaques, neurofibrillary tangles, mitochondrial dysfunction, and autophagy dysfunction are commonly used as the important indicators for evaluating the effects of potential candidate drugs. The rhizome of *Salvia miltiorrhiza* (known as ‘Danshen’ in Chinese), a famous traditional Chinese medicine, which is widely used for the treatment of hyperlipidemia, stroke, cardiovascular and cerebrovascular diseases. Increasing evidences suggest that the bioactive components of Danshen can improve cognitive deficits in mice, protect neuronal cells, reduce tau hyperphosylation, prevent amyloid-β fiber formation and disaggregation. Here we briefly summarize the studies regarding the effects of bioactive component from Danshen on those major characteristics of AD in preclinical studies, as well as explore the potential of these Danshen component in the treatment of AD.

## Background

**Fig. 1:**
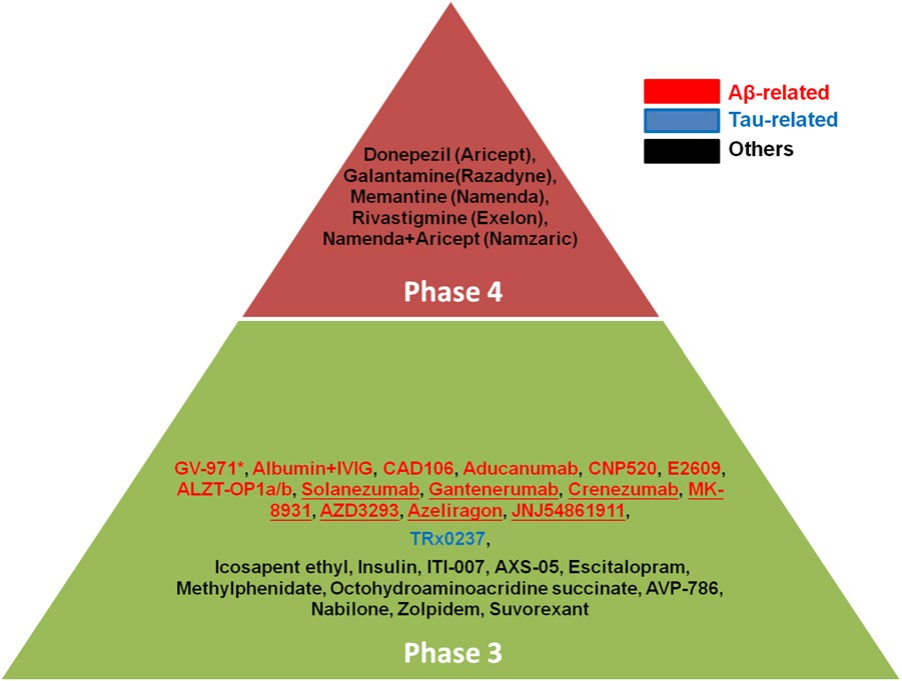
Phase 3 and 4 clinical trials in 2018. Asterisk, completed; Underline, failed

Alzheimer’s disease (AD) is the most common neurodegenerative disorder, which is characterized by the symptoms such as the progressive loss of memory and the cognitive impairment. The symptoms of AD result from the death or functional loss of neurons in the brain [1]. The incidence of AD increases as age over 65 years old; thus aging is commonly considered as the main risk factor. However, due to the poor understanding of etiology and pathogenic mechanisms in AD, the development of effective drugs still remains stagnant. At present, only four U.S. Food and Drug Administration (FDA)-approved drugs, including cholinesterase inhibitors (Aricept, Exelon, Razadyne) and memantine (Namenda) are able to temporarily reduce AD symptoms [2] (Fig. 1). The combination of Namenda and Aricept (Namzaric) is recently approved to improve the memory and the cognitive in patients with moderate to severe AD. Tacrine is the first cholinesterase inhibitor approved for the treatment of AD in 1993, but it is discontinued due to its strong hepatotoxicity [3]. It should note that these approved medicines cannot cure AD or slow AD process, they just help some symptoms for a limited time.

In addition to aging, genetic factors also involve in the pathogenesis of AD. Rare autosomal-dominant gene mutations cause familial AD (FAD) which exhibits the similar pathological and clinical features of AD except some cases are early-onset. Other risk factors such as smoking, air pollution and infection have been reported to involve in the pathogenesis of AD [4–6]. However, the crosstalk among these factors make the understanding of pathogenic mechanisms of AD become more difficult. Up to now, most AD studies propose the possible pathogenesis mechanisms based on the clinical characteristics of AD such as amyloid β (Aβ) plaques, neurofibrillary tangles (NFTs), mitochondrial dysfunction and autophagy dysfunction. These characteristics have been demonstrated to affect the functions and survival of neurons. Thus, these characteristics of AD are commonly used as the important indicators for evaluating the effects of potential candidate drugs.

The rhizome of *Salvia miltiorrhiza* (known as ‘Danshen’ in Chinese), a well-known traditional Chinese medicine, which is widely used for the treatment of hyperlipidemia, stroke, cardiovascular and cerebrovascular diseases [7–10]. Increasing studies suggest that the extraction from Danshen displayed the neuroprotectvie effects in various AD models. Total salvianolic acid from Danshen was found to reduce the learning and memory impairments in APPswe/PS1dE9 mice [11]. The aqueous extraction of Danshen could reduce Aβ-induced neurotoxicity in human neuroblastoma SH-SY5Y cells [12]. In addition, the extraction from Danshen was reported to enhance the differentiation of induced pluripotent stem cells (iPSCs)-derived neural stem cells (NSCs) into neurons in vitro, and improve the recovery function of transplanted NSCs in the rat ischemic brain in vivo [13]. The major components of Danshen such as salvianolic acid A, salvianolic acid B, danshensu, tanshinone I, tanshinone IIA, and cryptotanshinone exhibit the neuroprotective effects, which are attracting strong attention for the treatment of AD [14–17]. In this review, we briefly summarize the studies regarding the effects of Danshen components on the major characteristics of AD, and explore their possibility for the treatment of AD.

## The characteristics of AD

### Aβ plaques

The most well-known characteristic of AD is Aβ plaques [18–21]. Accumulation of Aβ plaques is positively correlated with the cognitive impairment in AD [22, 23]. Aβ is a polypeptide containing 37 to 49 amino acid residues, generated from its precursor amyloid precursor protein (APP) processing via cleavage by β-secretase and γ-secretase. In Aβ hypothesis, Aβ toxicity is considered as the primary cause of AD. Thus, anti-Aβ strategies to reduce Aβ toxicity or generation have been the major focus for the development of AD drugs. It is well known that γ-secretase inhibitors show a significant reduction of plasma Aβ levels in AD patients, but eventually fail in previous clinical trials [24]. Furthermore, in 2018, 14 Aβ-related candidate drugs still are in the phase 3 clinical trials [25]. However, up to now, over a half of them include anti-Aβ antibodies (Aducanumab, Solanezumab, Gantenerumab, and Crenezumab), β-secretase inhibitors (MK-8931, AZD3293, JNJ54861911), are known to fail in the phase 3 (Fig. 1), revealing that Aβ may be the consequence, not the pathogenic cause.

Aβ plaques is caused by the accumulation of extracellular Aβ, however why secreted Aβ accumulates in AD brain remains unknown. Increasing studies indicate that abnormal APP processing involves in the development of AD [26, 27]. The metabolism of APP is very rapid in neurons [28], APP or its metabolites such as the carboxyl-terminal fragment of APP (APP-CTF) and Aβ may be easy to accumulate once the APP processing is disrupted. Abnormal intracellular levels of APP or APP-CTF has been reported to cause tau pathology and autophagy dysfunction [27]. Thus, modulating or enhancing APP metabolism may be a potential strategy for anti-AD.

### NFTs

In addition to Aβ plaques, NFTs are commonly known as a major characteristic of AD [29]. NFTs are insoluble twisted fibers comprised of the accumulation of hyperphosphorylated tau protein, which are found inside AD neurons. Tau is a microtubule-associated protein that mediates the stability of tubulin assemblies. The phosphorylation of tau negatively regulates its activity in enhancing microtubule assembly [30]. Tau is phosphorylated by several kinases such as glycogen synthase kinase 3β (GSK3β), c-Jun N-terminal kinase (JNK), cyclin-dependent kinase 5 (Cdk5), extracellular signal-regulated kinase (ERK), and microtubule-associated regulatory kinase [31]. Increasing evidence supports the hyperphosphorylation caused by these tau-related kinases is a critical step in the accumulation of tau [32]. Thus, reducing the activities of tau-related upstream kinases to prevent the accumulation of hyperphosphorylated tau may be a therapeutic strategy for the treatment of AD.

### Mitochondrial dysfunction

Mitochondria is an important organelle for energy generation via mitochondrial respiratory chain. The damage in mitochondria triggers the loss of ATP and the increase of ROS, further resulting in apoptotic cell death. Mitochondrial dysfunctions such as the decreased mitochondrial membrane potential, the increased permeability, and the generation of excess reactive oxygen species (ROS) are found in the early stage of AD brain [33, 34], suggesting that mitochondrial dysfunction may involve in the loss of neurons in AD. Recently, the accumulation of APP and Aβ are found in the mitochondria of human AD brain, providing the clinical evidence to support that abnormal APP metabolism may be associated mitochondrial dysfunction and impaired energy metabolism [35, 36]. Since the critical role of mitochondria in neurodegeneration and neuronal death, how to prevent the mitochondrial dysfunction has been concerned in AD studies.

### Autophagy dysfunction

Autophagy is a catabolic process that delivers cytoplasmic organelles and substrates to lysosomes for degradation. It plays an important role in the turnover of organelles and proteins, the cellular energy balance as well as the cell survival [37–39]. Autophagy is a key regulator for Aβ generation and clearance, as well as mitochondria turnover [40]. Abnormal accumulation of autophagic vacuoles is found in AD brain. Autophagy defect phenotypes such as the lysosomal dysfunction, the impairment of autophagy degradation and the defect of mitophagy are found in neurons as well as non-neuronal cells from FAD patients [41–45]. Aβ secretion and plaque formation are reported to associate with autophagy dysfunction [46]. Thus, autophagy dysfunction is commonly considered as one of AD characteristics. Increasing studies indicate that the using of small molecular compounds to promote autophagy exhibits the promising effects on reducing Aβ, APP and tau pathology, even improving cognitive deficits [47–53], suggesting enhancing autophagy may be a potential strategy to reduce AD-related protein accumulation and mitochondrial dysfunction in the AD process.

## The effects of Danshen components on AD characteristics

### Salvianolic acid A

Salvianolic acid A is one of the most active components in Danshen, which displays the strong free radical scavenging ability due to its polyphenolic structure, as well as anti-apoptosis, and anti-inflammation [54, 55]. Aβ plaques comprise both Aβ40 and Aβ42. Compared with Aβ40, the longer Aβ42 is more easy to aggregate, and leads to more serious cognitive loss in animals [56]. In the study of Cao et al. [57], they found that salvianolic acid A (1, 4, 10, and 40 μM) could block the self aggregation of Aβ42. It (50 and 100 μM) also almost completely disaggregated Aβ42 pre-formed fibers. They used circular dichroism and molecular dynamic simulations to demonstrate that salvianolic acid A directly bind to the C-terminal of Aβ42 and stabilize α-helical conformations of Aβ42, contributing to its ability to prevent the aggregation of Aβ42. In addition, they found that salvianolic acid A (5, 10, 20, and 40 μM) was able to decrease Aβ42-induced neurotoxicity in SH-SY5Y cells. They also evaluated the anti-Aβ effect of salvianolic acid A using transgenic *C. elegans* strain CL4176 which over-expressed human Aβ42 in muscles for assaying Aβ-induced paralysis. They found that salvianolic acid A (50 and 200 μM) reduced total Aβ and Aβ-induced paralysis in these transgenic worms. These results suggest that salvianolic acid A may prevent Aβ-induced damage via reducing Aβ aggregation.

Total salvianolic acid extracted from Danshen is reported to reduce the learning and memory impairments in APPswe/PS1dE9 mice via decreasing Aβ42 and Aβ40 [11], hinting that salvianolic acid A may have the capability to regulate APP processing. β-Secretase is the key enzyme for APP processing to generate Aβ. In the study of Tu et al. [58], enzyme kinetic analysis showed that salvianolic acid A (IC_50_: 13 μM) was able to inhibit the activity of β-secretase. Their computer docking analysis predicted that salvianolic acid A bound tightly to the active site of β-secretase. However, no further cell-based study provides the evidence to support the ability of salvianolic acid A to regulate β-secretase.

GSK3β is considered as a possible therapeutic target against AD because its function involves in phosphorylation of tau, mitochondria function and cell survival [59, 60]. Through enzyme kinetic assay, Paudel et al. found that salvianolic acid A (IC_50_: 30 μM) exhibited the inhibitive effect on the activity of GSK3β [61]. However, It still lacks of the direct evidence to support the effects of salvianolic acid A on GSK3β-related events.

### Salvianolic acid B

Salvianolic acid B is the major and most active antioxidant from Danshen, which can prevent cells from Aβ-induced cytotoxicity. He et al. reported that salvianolic acid B (50 μM) reduced mitochondrial stress and preserved synaptic density in Aβ42-treated primary cultured mouse neurons [62]. In addition, salvianolic acid B (10, 100, and 200 μg/ml) could protect PC12 cells against Aβ (25–35)-induced increase of Ca^2+^-intake and LDH release [63]. In addition to anti-Aβ toxicity, salvianolic acid B also shows the ability to regulate APP processing. Tang et al. reported that salvianolic acid B (50 and 100 μM) was able to reduce the levels of Aβ40, Aβ42 and ROS in the culture media of SH-SY5Y cells with overexpression of SwedAPP [64]. They found that salvianolic acid B was able to affect the metabolism of APP in these cells. Salvianolic acid B reduced the level of secreted APPβ via down-regulating the expression of β-secretase, whereas the level of sAPPα was increased by treatment of salvianolic acid B to up-regulate the expression of α-secretase. In the study of Durairajan et al. they found that salvianolic acid B could decrease the generation of Aβ in N2a-mouse and H4-human neuroglioma cell lines expressing SwedAPP [65]. They got the identical results with Tang et al, but did not observe that salvianolic acid B affected the activities of α-secretase and γ-secretase. They used computer docking analysis to predict salvianolic acid B may interact with β-secretase [65], suggesting that salvianolic acid B may directly modulate β-secretase activity. However, in the study of Tu et al. [58], enzyme kinetic analysis showed that salvianolic acid B could not inhibit the activity of β-secretase. These results provide strong evidence to demonstrate that salvianolic acid B is able to reduce amyloidogenic pathways via down-regulating the expression of β-secretase, and increases the activity of α-secretase which cleave APP in non-amyloidogenic pathways. This activity of salvianolic acid B promote APP processing toward the non-amyloidogenic generation, may provide an alternative way for reducing Aβ generation.

In Paudel et al. study, salvianolic acid B (IC_50_: 7 μM) could block GSK3β activity in enzyme kinetic assay [61]. Their computer docking analysis predicted that salvianolic acid B could bind to the catalytic domain of GSK3β, suggesting it might be an ATP-competitive inhibitor of GSK3β. In addition, salvianolic acid B (25, 50, and 100 μM) also was found to reduce the activity of GSK3β in SH-SY5Y cells with overexpression of APP mutant [64]. The ability of salvianolic acid B to blocking the activity of GSK3β in vitro may prevent tau from hyperphosphorylation, but further investigation is needed.

NFE2 p45-related factor 2 (Nrf2) plays a regulatory role in the expression of genes involved in mitochondria biogenesis and intracellular ROS scavenging, which may confer the protection of mitochondria. Zhou et al. found that salvianolic acid B (10, 50, and 100 μM) could enhance the intracellular antioxidant defense mechanism involving Nrf2-induced antioxidant enzymes in mouse midbrain neuron-glia cultures [66]. In addition, salvianolic acid B was able to decrease Aβ-induced mitochondrial stress in primary cultured mouse neurons [62]. These results suggest the salvianolic acid B may provide the protection to mitochondria.

Salvianolic acid B could work as a novel autophagy inducer in non-neuronal cells [67, 68]. However, in brain, only Jiang et al. reported that the intraperitoneally injection with 20 mg/kg salvianolic acid B reduced lipopolysaccharide (LPS)-induced the increase of autophagic markers and neuroinflammation, thereby resulting in neuroprotective in the brain of rats [69]. However, LPS model is not related to AD, cannot support the autophagy-regulating effects of salvianolic acid B in autophagy dysfunction in AD.

### Danshensu

Danshensu is an active component of Danshen with wider cardiovascular effects. Danshensu was also reported to provide neuroprotection in the neurotoxin-induced injury model, and could pass the blood-brain barrier (BBB) of rats, suggesting that danshensu has the potential in the treatment of brain disorders [54, 70]. In addition, danshensu (10, 100, and 200 μg/ml) could attenuate Aβ (25–35)-induced increase of Ca^2+^-intake and LDH release in PC12 cells [63]. Previous study indicated that danshensu (100, 200, 400 μM) alone was able to enhance the intracellular antioxidant defense mechanism involving Nrf2-induced antioxidant enzyme heme oxygenase 1, thereby provided the protection against 6-OHDA-induced oxidative damage in PC12 cells [71]. These results hint that danshensu may have the positive effects on mitochondrial function and cell survival. However, there are no further reports regarding the effects of danshensu on other characteristics of AD.

### Tanshinone I

Tanshinone I is a bioactive lipophilic compound isolated mainly from Danshen. Tanshinones was reported to exhibit antioxidant and anti-inflammatory effects in the ischemic injury models [72, 73]. In the study of Wang et al, they found that tanshinone I (20 and 40 μM) could reduce the formation of Aβ42 fibrils and disassemble Aβ42 aggregation [74]. Tanshinone I (4 μM) also provided the protection against Aβ-induced cytotoxicity in SH-SY5Y cells. The molecular docking predicted that tanshinone I had the higher affinity with the structure of Aβ. However, the working concentration of anti-Aβ aggregation of tanshinone I is not consistent with its protective effects.

In the study of de Oliveira et al., they indicated that tanshinone I (2.5 μM) was able to confer mitochondrial protection such as reducing mitochondrial toxin-induced impairments of complex I and mitochondrial membrane potential in SH-SY5Y cells [75]. Their results demonstrated that the treatment with tanshinone I alone could up-regulate antioxidant enzymes, such as Mn-superoxide dismutase, glutathione peroxidase, and both catalytic and modifier subunits of γ-glutamate-cysteine ligase via enhancing the intracellular antioxidant defense mechanism of Nrf2, revealing that tanshinone I has the ability to maintain the mitochondria functions via increasing the expression of Nrf2.

### Tanshinone IIA

Tanshinone IIA is one kind of tanshinones extracted from Danshen, which exhibits the antioxidant and anti-inflammatory activities. The effects of tanshinone II on the Aβ-related events have been reported. Shi et al. indicated that pretreatment of tanshinone IIA (10, 20, and 40 μM) protected primary cortical neurons from Aβ25–35 induced neurotoxicity [76]. They found that tanshinone IIA reduced Aβ-induced the cleavage of p35 into p25 and thus inhibited the Cdk5 pathway, suggesting that blocking the p35/Cdk5 pathway may contribute to the protective effects of tanshinone IIA. Liu et al. also found that tanshinone IIA (0.1, 1, and 10 μM) reduced Aβ-induced oxidative stress and apoptosis in rat cortical neurons by inhibiting lipid peroxidation and ROS increase, stabilizing mitochondrial membrane potential, as well as reducing cytochrome c release from mitochondria [77]. The protective effects of tanshinone IIA on SH-SY5Y cells against Aβ42-induced cytotoxicity was reported by Wang et al. [74] and Yang et al. [78]. In addition to the different working concentrations, the main difference is that Wang et al. reported the protective effects of tanshinone IIA likes tanshinone I resulted from the suppression of Aβ42 fibrils formation and the disassembly Aβ42 aggregation via directly binding to Aβ, whereas Yang et al. found that reducing Aβ42-induced endoplasmic reticulum stress contribute to the protective effects of tanshinone IIA. In animal study, Maione et al. indicated that tanshinone IIA (10 mg/kg) reduced memory decline and the increase of neuroinflammatory markers in Aβ42-injected mice [79]. These results show that the multiple mechanisms involve in the protective effects of tanshinone IIA against Aβ toxicity.

Tanshinone IIA was reported to reduce Aβ-induced the activation of tau-related kinase Cdk5, thereby attenuate the expression of phosphorylated tau in primary cortical neurons [76]. Tanshinone IIA also plays as a Nrf2 inducer in various cells [80, 81]. In SH-SY5Y cells, tanshinone IIA (5, 10, and 20 μg/ml) could induce the expression of NRF2 binding site-regulated genes, thereby provided the neuroprotection against neurotoxin 6-OHDA [82]. Zhu et al. reported that tanshinone IIA (0.2, 1, 2 and 5 μg/ml) also protects hippocampal neuronal cells HT-22 from ischemic damages such ROS increase, abnormal autophagy induction, and mitochondrial impairment via enhancing PI3K/Akt/mTOR signals [83]. These abilities of tanshinone IIA may bring the benefit to reduce the characteristics in AD brain.

### Cryptotanshinone

Cryptotanshinone also is one kind of tanshinones. Several studies suggest that the activities of cryptotanshinone involved in reducing the Aβ aggregation and toxicity, as well as up-regulating α-secretase. Mei et al. reported that cryptotanshinone (1, 2.5, and 5 μM) could inhibit Aβ42 spontaneous aggregation and (5 and 10 μM) dramatically reduced Aβ42-induced cell apoptosis and ROS increase in SH-SY5Y cells [84]. In addition, cryptotanshinone (3 and 10 mg/kg) has been reported to reduce memory decline and neuroinflammation in Aβ42-injected mice [79], supporting the anti-Aβ ability of cryptotanshinone. The abnormal processing of APP is one of Aβ-related events in AD patients [85]. Met et al. reported that cryptotanshinone (15 mg/kg) strongly attenuated amyloid plaque deposition and the decease of cognitive ability in APP/PS1 transgenic mice [86]. Interesting, their further study found that cryptotanshinone was able to enhance PI3K-mediated the expression of α-secretase which cleave APP in non-amyloidogenic pathways [87]. This effect of cryptotanshinone on promoting APP processing toward the non-amyloidogenic generation, may provide an alternative way for reducing Aβ generation.

## Discussion

Aβ plaques, NFTs, mitochondrial dysfunction, and autophagy dysfunction are the characteristics of AD, which may be crucial indicators for evaluating the pharmaceutical effects of promising AD drugs. In this review, we evaluate the effects of six components from Danshen on these major characteristics of AD (Table 1). In these preclinical studies, each components are able to reduce Aβ toxicity (Table 2). Salvianolic acid A, tanshinone I, tanshinone IIA, and cryptotanshinone show the protective activities against Aβ-induced cell damage as well as reduced Aβ aggregation. Compared their working concentration in anti-Aβ-induced cytotoxicity and anti-Aβ aggregation, the working concentrations of salvianolic acid A and cryptotanshinone in both activities are similar, suggesting that the protective effects of salvianolic acid A and cryptotanshinone against Aβ-induced cytotoxicity mainly result from its ability to reduce Aβ aggregation. However, due to the failure of most anti-Aβ therapies in clinical trials, anti-Aβ toxicity is not considered as one of the indicators. On the other hand, the ability of salvianolic acid B and cryptotanshinone affects APP processing via regulating the expression of secretases, may bring the benefit in reducing the Aβ formation.

**Table 1:** The effects of bioactive components from Danshen on major characteristics of AD

Compounds from Danshen	The effects on the characteristics of AD
Salvianolic acid A	Inhibit Aβ aggregation and disaggregates Aβ fibrils [47] Anti-Aβ-induced cytotoxicity [47] Inhibit the activity of β-secretase in enzyme kinetic assay [48] Inhibit the activity of GSK3β in enzyme kinetic assay [51]
Salvianolic acid B	Anti-Aβ-induced cytotoxicity [52, 53] Reduce Aβ generation in cells [54, 55] Down-regulate the expression of β-secretase in cells [54] Up-regulate the expression of α-secretase in cells [54] Inhibit the activity of GSK3β in enzyme kinetic assay [51] Reduce the activity of GSK3β in cells [54] Activate NRF2-mediated intracellular antioxidant defense mechanism [56] Reduce abnormal increase of autophagy in in vivo [59]
Danshensu	Anti-Aβ-induced cytotoxicity [53] Activate NRF2-mediated intracellular antioxidant defense mechanism [61]
Tanshinone I	Inhibit Aβ aggregation and disaggregates Aβ fibrils [64] Anti-Aβ-induced cytotoxicity [64] Activate NRF2-mediated intracellular antioxidant defense mechanism [65]
Tanshinone IIA	Inhibits Aβ aggregation and disaggregates Aβ fibrils [64] Anti-Aβ-induced cytotoxicity [64, 66–68] Reduce Aβ-induced memory decline and neuroinflammation in in vivo [69] Reduce Aβ-induced p35/Cdk5 pathway [66] Activate NRF2-mediated intracellular antioxidant defense mechanism [72] Reduce abnormal increase of autophagy in in vitro [73]
Crytotanshinone	Inhibits Aβ aggregation [74] Anti-Aβ-induced cytotoxicity [74] Reduce Aβ-induced memory decline and neuroinflammation in in vivo [69] Attenuate amyloid plaque deposition and the decrease of cognitive ability in APP/PS1 transgenic mice [76] Up-regulate the expression of α-secretase in cells [77]

**Table 2:** Comparison with the working concentration of components from Danshen in anti-Aβ-induced cytotoxicity and anti-Aβ aggregation

Compounds from Danshen	The effective concentration against Aβ-induced cytotoxicity	The effective concentration on Aβ aggregation
Salvianolic acid A	5, 10, 20, and 40 μM in SH-SY5Y cells [47]	1, 4, 10, and 40 μM [47]
Salvianolic acid B	10, 100, and 200 μg/ml in PC12 cells [53] 50 μM in mouse neurons [52]	–
Danshensu	10, 100, and 200 μg/ml in PC12 cells [53]	–
Tanshinone I	4 μM in SH-SY5Y cells [64]	20 and 40 μM [64]
Tanshinone IIA	4 μM in SH-SY5Y cells [64] 1, 5, 10, 20 μM in SH-SY5Y cells [68]	20 and 40 μM [64]
	10, 20, and 40 μM in mouse cortical neurons [66] 0.1, 1, and 10 μM in rat cortical neurons [67]	
Cryptotanshinione	5 and 10 μM in SH-SY5Y cells [74]	1, 2.5, and 5 μM [74]

Salvianolic acid B is able to directly inhibit the activity of tau-related kinase GSK3β, may confer the decrease in hyperphosphorylation of tau in AD. Tanshinone IIA and cryptotanshinone affect Aβ-induced upstream kinases such as Cdk5 and p38, thereby reduce the expression of hyperphosphorylated Tau. It is still unknown whether exogenous Aβ is the cause for tau pathology in the AD process. Most studies based on Aβ hypothesis may not accurately recapitulate the key aspects of AD. Thus, the further studies are needed to demonstrate their positive effects on tau pathology in suitable models.

Salvianolic acid B, danshensu, tanshinone I, and tanshinone IIA, are able to activate Nrf2 defense mechanism or reduce mitochondria-dependent apoptosis pathway which is critical to maintain mitochondrial functions under cell damage. Striking, salvianolic acid B and tanshinone IIA are reported to reduce abnormal autophagy in non AD model. Compared with other components, salvianolic acid B reduces four characteristics of AD, which shows more potential for the treatment of AD.

## Conclusion

Up to now, since the exact pathogenic mechanisms of AD are still poorly understood, thereby no any effective cures for slowing or preventing AD process. The bioactive components of Danshen confer the different positive effects on APP processing, tau hyper-phosphorylation, mitochondria dysfunction, as well as abnormal autophagy, further suggesting their potential in the treatment of AD. Among them, salvianolic acid B shows more potential because it appears to reduce four characteristics of AD in preclinical studies, supporting that the further development of salvianolic acid B is warranted, as a potential neuroprotectant with the multiple effects to reduce neuronal death in AD development.

## Data Availability

Not applicable.

## Abbreviations

Aβ: amyloid β
AD: Alzheimer’s disease
APP: amyloid precursor protein
APP-CTF: carboxyl-terminal fragment of APP
BBB: blood–brain barrier
Cdk5: cyclin-dependent kinase 5
ERK: extracellular signal-regulated kinase
JNK: c-Jun N-terminal kinase
FDA: Food and Drug Administration
FAD: familial Alzheimer’s disease
GSK3β: glycogen synthase kinase 3β
iPSCs: induced pluripotent stem cells
LPS: lipopolysaccharide
NFTs: neurofibrillary tangles
NRF2: NFE2 p45-related factor 2
NSCs: neural stem cells
ROS: reactive oxygen species
